# Effects of dietary hatchery by-products on growth performance, relative organ weight, plasma measurements, immune organ index, meat quality, and tibia characteristics of broiler chickens

**DOI:** 10.5713/ab.20.0755

**Published:** 2021-01-04

**Authors:** Won Jun Choi, Jong Hyuk Kim, Gi Ppeum Han, Chan Ho Kwon, Dong Yong Kil

**Affiliations:** 1 Department of Animal Science and Technology, Chung-Ang University, Anseong 17546, Korea

**Keywords:** Animal Protein Ingredient, Broiler Chicken, Fish Meal, Growth Performance, Hatchery By-products, Infertile Eggs

## Abstract

**Objective:**

The objective of the current study was to investigate the effects of dietary hatchery by-products (HBPs) as a replacement of fish meal (FM) on growth performance, relative organ weight, plasma measurements, immune organ index, meat quality, and tibia characteristics of broiler chickens.

**Methods:**

A total of 720 broiler chickens (3 d of age) were randomly allotted to 1 of 9 treatments with 8 replicates. Each replicate consisted of 5 male and 5 female birds. The basal diet was formulated to contain 5.0% commercial FM, whereas eight treatment diets were prepared by replacing 25%, 50%, 75%, or 100% of FM in the basal diet with infertile eggs (IFE) or a mixture of various HBPs (MIX); therefore, the inclusion levels of IFE or MIX in the experimental diets were 1.25%, 2.50%, 3.75%, or 5.00%. The diets and water were provided on an *ad libitum* basis for 32 d.

**Results:**

Increasing inclusion levels of IFE as a replacement of FM in diets had no effects on growth performance, plasma measurements, immune organ index, and tibia characteristics of broiler chickens. Increasing inclusion levels of IFE in diets increased (linear, p<0.05) meat lightness (L*) but decreased (linear, p<0.05) meat redness (a*). The breast meat pH at 1-h postmortem was increased (linear, p<0.05) by increasing inclusion levels of IFE in diets. Likewise, increasing inclusion levels of MIX in diets had no effects on growth performance, relative organ weight, plasma measurements, immune organ index, and tibia characteristics. However, increasing inclusion levels of MIX in diets increased (linear, p<0.05) 1-h postmortem pH but decreased (linear, p<0.05) 24-h postmortem pH of breast meat. Increasing inclusion levels of MIX in diets decreased (linear, p<0.05) thiobarbituric acid reactive substances values of breast meat.

**Conclusion:**

Both IFE and MIX are suitable alternatives to FM as protein ingredients in broiler diets.

## INTRODUCTION

Poultry diets are formulated primarily with various vegetable ingredients, which are relatively cheap but contain the less bioavailable nutrients than animal-derived ingredients and purified nutrients. Therefore, despite their high price, animal protein sources such as fish meal (FM) are often added to poultry diets, especially for broiler chickens, to satisfy the essential amino acid (AA) requirements. Feed cost constitutes the largest portion of the total cost of poultry production, and therefore, the identification and increased use of potential and inexpensive animal protein sources would significantly improve economic outcomes of the poultry industry [1].

The Korean poultry industry produces more than 4,000 tons of hatchery by-products (HBPs) every year. These HBPs include infertile eggs (IFE), unhatched eggs (UHE), low grade and dead chicks (LDC), and eggshells (ES) [2]. In commercial hatcheries, IFE are typically collected at the middle of the hatching process (e.g., examination of fertilization), whereas UHE, LDC, and ES are mostly collected at the end of the hatching process. The collected UHE, LDC, and ES are mixed to produce the final HBPs with or without inclusion of IFE. Currently, most HBPs produced in South Korea are composted as a fertilizer, and the raising environmental concerns necessitate an alternative solution for the use of HBPs. The HBPs contain high amounts of available nutrients such as AAs, calcium (Ca), and phosphorus (P) for animals. Several previous experiments have reported that the HBPs can be used as economical and potential protein ingredients for poultry diets [1,3]. However, few studies have directly evaluated a mixture of HBPs (MIX) as an alternative to FM, which is widely used as an animal protein ingredient in poultry diets. In addition, IFE can be collected separately from MIX, and thus, IFE is likely another potential source of animal protein. However, there is very limited information regarding the nutritive value of IFE and MIX in broiler diets, especially compared with FM.

Accordingly, the objective of the present study was to investigate the effects of dietary IFE and MIX (a conventional mixture of IFE, UHE, LDC, and ES) as a replacement of FM on growth performance, relative organ weight, plasma measurements, immune organ index, meat quality, and tibia characteristics of broiler chickens.

## MATERIALS AND METHODS

### Animals, experimental design, and diets

The protocol for the current experiment was reviewed and approved by the Institutional Animal Care and Use Committee at Chung-Ang University (IACUC No. 2018-00136). In total, 720 Ross 308 broiler chicks (1 d of age) were obtained from a local hatchery (Dongsan broiler hatchery, Cheonan, Korea). The chicks were fed a commercial pellet starter diet until 3 d of age. At the 3 d of age, chicks with an average initial body weight (BW) of 82±2.1 g were allotted to 1 of 9 treatments with 8 replicates in a completely randomized design. Each replicate consisted of 5 male and 5 female birds.

The HBPs, including IFE, UHE, LDC, and ES, were separately obtained from a local hatchery (Dongsan broiler hatchery, Cheonan, Korea). A mixture of assorted HBPs (designated MIX) was produced with a composition of 55% IFE, 10% UHE, 10% LDC, and 25% ES, which was based on the consideration of typical production ratio of each HBP in the commercial hatchery [2]. The IFE and MIX samples were ground using a meat chopper (MN-225, Hankook Fujee, Hwaseong, Korea) and dried at 50°C for 24 h in a vertical convention oven (LDO-630F, Daihan Labtech, Namyangju, Korea) to ensure that they contained less than 2.0% water. The commercial FM was also prepared for diet formulation. The analyzed nutrient concentrations of IFE, MIX, and FM are presented in Table 1.

The basal diet for the control group was formulated to contain 5.0% FM, which is close to the maximum inclusion levels of FM in the commercial broiler diet. Eight treatment diets were prepared by replacing 25%, 50%, 75%, or 100% of FM with either IFE or MIX. Therefore, the actual inclusion levels of IFE or MIX in the experimental diets were 1.25%, 2.50%, 3.75%, or 5.00%. All nutrient concentrations of 9 treatments diets (Tables 2, 3) were formulated to be identical and satisfied for nutrient requirements of growing and finishing broiler chickens [4]. The experimental diets and water were provided on an *ad libitum* basis for 32 d from 3 d to 35 d of age. The room temperature was maintained at 30°C at the start of the experiment and then gradually decreased to 24°C as recommended by the Ross manual [4]. A 23-h lighting schedule was applied during the experimental period. The body weight gain (BWG) and feed intake (FI) were measured when the chickens were 35 d old. The mortality was recorded daily. The feed conversion ratio was calculated by dividing FI (kg) by BWG (kg) after correcting for mortality.

### Sample collection and analysis

At the end of experiment (35 d of age), the individual weights of all broiler chickens were recorded. The broiler chickens, which is the closest to average weight of the replicated cage, were euthanized via CO_2_ asphyxiation. Blood samples from each bird were collected via heart puncture into a 10-mL vacutainer tube containing sodium heparin (BD, Franklin Lakes, NJ, USA) and then centrifuged at 3,000×g at 4°C for 20 min to obtain the plasma. The plasma samples were stored at −20°C before further analysis. The concentrations of albumin, alanine aminotransferase (ALT), aspartate aminotransferase (AST), creatinine, glucose, total protein, and total glyceride in the plasma were analyzed using a HITACHI 7020 automatic analyzer (Hitachi, Tokyo, Japan).

The liver, kidney, small intestine, and gizzard were collected, and their relative weight (the weight as a percentage of the total BW) were calculated. The immune organs such as thymus, spleen, and bursa of Fabricius were also collected and weighed. The values for immune organ index were calculated by the following equation: (immune organ weight [g]/BW [g])×1,000 [5].

The right portion of the breast meat was detached from the sternum for meat quality analyses. The color of breast meat was measured directly postmortem using a colorimeter (model CR-10, Konica Minolta, Tokyo, Japan) with the lightness (L*), redness (a*), and yellowness (b*) values of the meat surface being recorded. The breast meat was then weighed and divided into 4 parts, which were respectively used to measure pH at 1 h and 24 h postmortem, water holding capacity (WHC) at 24 h postmortem, and thiobarbituric acid reactive substances (TBARS) at 7 d postmortem. The 4 pieces of breast meat were stored at 4°C until further analysis. The meat pH was measured using a pH meter (Hanna Instruments, Nusfalau, Romania) at a depth of 1 cm. The WHC of breast meat was measured at 24 h postmortem according to the method described by Lee et al [6]. A portion of meat samples (1.5 g) was centrifuged at the speed of 3,000×g at 5°C for 15 min. The WHC (%) was calculated as ([weight before centrifugation – weight after centrifugation]/weight before centrifugation)×100 [6]. The TBARS values of breast meat were measured as an indicator of lipid oxidation, following the method described by Lee et al [6]. Firstly, 5 g of the meat sample were placed in a 50-mL test tube with 15 mL of deionized distilled water and then homogenized in ice using an electric homogenizer (Tissue Tearor; Biospec, Bartlesville, OK, USA). Then, 1 mL of homogenized solution was placed in a disposable test tube (13 mm ×100 mm) with 2 mL of thiobarbituric/trichloroacetic acid and 50 μL of 100 mL/L butylated hydroxyanisole. The mixture was incubated in boiling water for 15 min to develop the color. The mixture was cooled in an ice bath for 10 min and then vortexed. The mixture was centrifuged at 2,000×g for 15 min, and then the supernatant was collected. Finally, the absorbance of the supernatant at 531 nm was determined using a microplate reader (Spectramax 190, Molecular Devices, Sunnyvale, CA, USA).

Both right and left tibias were collected to analyze bone characteristics. The muscle attached to the tibias was removed. The concentrations of Ca, P, and ash in the right tibia were analyzed, whereas the left tibia was used to measure the breaking strength. The right tibia was dried at 100°C for 24 h in a drying oven and then placed in a Soxhlet apparatus for 48 h to remove the fat using ethyl ether as a solvent. Afterwards, the right tibia was dried again at 100°C for 24 h in a drying oven and then ground to powder. Ground samples were ashed in a muffle furnace at 600°C for 24 h to measure tibia ash concentrations [7,8]. The Ca and P concentrations in the ash of the right tibia were measured using an inductively coupled plasma spectrometer (Optima 5300 DV, Perkin Elmer, Shelton, CT, USA) as described by Kurtoğlu et al [9]. Breaking strength of the left tibia was analyzed using a TAHDi texture analyzer (Stable Micro Systems, Surrey, UK) according to the method described by Shaw et al [10].

### Statistical analysis

All data were analyzed using analysis of variance in a completely randomized design using the MIXED procedure (SAS Institute Inc., Cary, NC, USA). All data were checked for outliers and normal distribution using the UNIVARIATE procedure of SAS (SAS Institute Inc., USA). One outlier was identified and removed from the final data analysis. The replicate was used as an experimental unit for all measurements. There were 9 experimental diets in this study, including 1 basal diet, 4 diets containing IFE, and 4 diets containing MIX. In order to evaluate individual IFE and MIX as alternatives to FM, IFE and MIX treatments were analyzed separately, with each evaluation involving 5 experimental diets (1 basal diet and 4 treatment diets containing different inclusion levels of IFE or MIX). The LSMEANS procedure was used to calculate treatment means and the PDIFF option of SAS was used to separate the means if the difference was significant. Orthogonal polynomial contrasts test was also used to determine the linear and quadratic effects of increasing inclusion levels of IFE or MIX as a replacement of FM in diets. Significance for statistical tests was set at p<0.05.

## RESULTS AND DISCUSSION

### Nutrient composition of infertile eggs and hatchery by-product mixture

The proximate and AA compositions of the IFE and MIX used in this experiment are presented in Table 1. The concentrations of crude protein (CP), Ca, and P in the MIX were comparable to previous results of a mixture of HBPs [11]. However, other studies have used HBP mixtures with different ratios of each HBP, leading to differences in nutrient concentrations comparing with HBPs used in this study [12]. The concentrations of CP and P were similar between IFE and MIX, whereas MIX had a greater concentration of Ca than IFE because of the inclusion of UHE, LDC, and ES, which contained high amounts of Ca. Based on the concentrations of CP and Ca, both IFE and MIX can be good sources of protein and Ca for broiler diets.

### Growth performance

Increasing inclusion levels of IFE or MIX from 0% to 5.0% as a replacement of FM in diets had no effects on growth performance of broiler chickens (Tables 4, 5), which indicates that both IFE and MIX are suitable replacements of FM in broiler diets if the diets are formulated to keep the constant energy and nutrient concentrations. A similar result was observed by Rasool et al [13] who used a control diet containing 12% FM and found that 100% replacement of FM with HBPs had no adverse effects on growth performance of broiler chicks. The reason for these results is that both FM and HBPs (i.e., IFE and MIX) are animal protein sources and contain high amounts of available essential AAs and minerals for broiler chickens [14].

### Plasma measurements

Plasma measurements, including albumin, ALT, AST, creatinine, glucose, total protein, and total glyceride were not influenced by increasing inclusion levels of IFE or MIX as a replacement of FM in broiler diets (Tables 6, 7). The plasma concentrations of albumin, ALT, and AST have been routinely used for monitoring the liver health because those concentrations are increased when liver cells are damaged [15,16]. In addition, the plasma concentrations of creatinine are used for a biomarker for the renal health because creatinine in the body is mainly excreted through the kidney [17]. The plasma concentrations of glucose, total protein, and total glycerides observed in the current experiment fell within the normal range for poultry. Therefore, both IFE and MIX appear to have no negative effects on liver and kidney functions as well as nutrient metabolism in broiler chickens.

### Relative organ weight

Increasing inclusion levels of IFE as a replacement of FM in diets had no effects on the relative weight of the breast, liver, kidney, and small intestine. The relative weight of the gizzard was increased (quadratic, p<0.05) with increasing inclusion levels of IFE in the diets (Table 8). However, increasing inclusion levels of MIX had no effects on the relative weight of any of the measured organs (Table 9).

The current observations are in line with a previous study reporting no difference in breast weights among birds fed diets containing from 0% to 4.5% HBPs [18]. Likewise, Abiola et al [11] also reported that the relative liver and gizzard weights were not affected by replacing FM with HBPs in broiler diets. In addition, the values of relative weights of breast, liver, kidney, and small intestine as observed in this study fell in the normal range reported previously [19–22]. However, interestingly we found a quadratic association between increasing inclusion levels of IFE in diets and the relative gizzard weight, and the reason for this observation is not clear. Despite this unexpected result, the values for the relative gizzard weight as observed in the current experiment also remained within the normal range from 1.2% to 1.5% of BW [23,24], and no abnormal enlargement in the gizzard size was identified. Thus, increasing inclusion levels of IFE or MIX up to 5.0% as a replacement of FM in diets are unlikely to lead to considerable changes in the gastrointestinal growth and development of broiler chickens.

### Immune organ index

There were no linear or quadratic effects of increasing inclusion levels of IFE or MIX as a replacement of FM in diets on the immune organ index (Tables 10, 11). The thymus is responsible for T-lymphocyte differentiation and proliferation, whereas B-lymphocytes is matured in the bursa of Fabricius [25,26]. The spleen plays a role in the regulation of cellular and humoral immunity in poultry via activation of T-lymphocytes, B-lymphocytes, and macrophage [27]. Therefore, the relative development of the thymus, bursa of Fabricius, and spleen can be used to evaluate immune function and health status of poultry [28,29]. Therefore, our results may indicate that replacing FM with IFE or MIX up to 5.0% in diets has no adverse effects on immune function of broiler chickens.

### Meat quality

The WHC and TBARS values of breast meat were not influenced by increasing inclusion levels of IFE as a replacement of FM in diets (Table 12). However, increasing inclusion levels of IFE in diets increased (linear, p<0.05) meat lightness (L*) but decreased (linear, p<0.05) meat redness (a*). The 1-h postmortem pH of breast meat was increased (linear, p<0.05) by increasing inclusion levels of IFE in diets. In particular, the pH was significantly greater (p<0.05) in birds fed diets containing 3.75% IFE than in birds fed the basal diet. However, the pH at 24 h postmortem was not affected by replacing FM with IFE.

Increasing inclusion levels of MIX in diets did not influence meat color and WHC of breast meat (Table 13). However, increasing inclusion levels of MIX in diets increased (linear, p<0.05) pH at 1 h postmortem but decreased (linear, p<0.05) pH at 24 h postmortem. In addition, increasing inclusion levels of MIX in diets decreased (linear, p<0.05) TBARS values of breast meat.

Meat color is one of the important factors affecting consumers’ preferences with regard to broiler meat [6]. For broiler breast meat, L* values of 42 to 50 and a* values of 1.9 to 5.3 have been widely reported [30–32]. The L* and a* values observed in this experiment fell within the quantitative range although the linear increase in L* value and linear decrease in a* value were observed with increasing inclusion levels of IFE in this experiment. It is not clear whether some specific chemical compounds in eggs (i.e., IFE) may affect breast meat colors, as no such effect has been reported previously in broiler chickens. However, it appears that consumers would be unable to distinguish HBP-fed birds from FM-fed birds on the basis of meat color.

The low pH value of breast meat is often associated with low meat quality [33]. In the current study, the pH values at 1 h postmortem were increased (linear, p<0.05) by increasing inclusion levels of both IFE and MIX, whereas pH values at 24 h postmortem were decreased (linear, p<0.05) with increasing inclusion levels of MIX. Postmortem pH is dependent primarily on the amount of glycogen in the muscle and the rate of its degradation to lactic acid [34]. However, it is unclear whether some specific chemical compounds in IFE and MIX may influence the concentration or degradation rate of glycogen in the muscle. However, all pH values of breast meat observed in this experiment fell within the normal range from 6.20 to 6.54 for 1 h postmortem [21,22] and from 5.85 to 6.34 for 24 postmortem [21,32], indicating that replacement of FM with IFE and MIX exerts no adverse effects on the postmortem pH of breast meat.

The TBARS value is used as an indicator of lipid peroxidation, and thus meat with low TBARS values is considered less oxidized [6]. Interestingly, we found that increasing replacement of FM with both IFE and MIX tended to decrease TBARS values of breast meat although the significance was only detected for broiler chickens fed diets containing MIX. Thus, replacement of FM with IFE and MIX is likely to decrease lipid peroxidation in breast meat. This observation is likely caused by the fact that FM contains a greater amount of polyunsaturated fatty acids as compared to other animal protein sources [35], as it is well-known that polyunsaturated fatty acids are more prone to oxidation than saturated fatty acids [36]. Therefore, one potential benefit of replacing FM with IFE or MIX may be the improvement in lipid stability of breast meat, which can increase the shelf-life of breast meat. It should be noted, however, that all TBARS values measured in this experiment fell within the normal range from 0.18 to 1.32 for 7 d postmortem [21,37]. Therefore, it appears that neither FM nor both HBPs (i.e., IFE and MIX) have considerable effects on lipid stability of the breast meat.

### Tibia characteristics

The tibia characteristics, including concentrations of ash, Ca, and P, and breaking strength were not affected by increasing inclusion levels of IFE or MIX (Tables 14, 15). This is likely related to the fact that all diets were formulated to contain the identical concentrations of minerals such as Ca and P. In addition, minerals such as Ca and P in the IFE and MIX are mostly derived from the ES, and it has been reported that Ca and P present in the ES are highly digestible and utilizable by poultry [2].

## CONCLUSION

Replacement of FM with HBPs as IFE and MIX up to 5.0% in diets has no adverse effects on growth performance, plasma measurements, relative organ weight, immune organ index, breast meat quality, and tibia characteristics of broiler chickens, provided that diets are formulated with appropriate energy and nutrient concentrations. One potential benefit of replacing FM with IFE or MIX may be the improvement in lipid stability of broiler meat by decreasing concentrations of polyunsaturated fatty acids. Therefore, both IFE and MIX can serve as economically feasible alternatives to FM as a protein source in broiler diets.

## Figures and Tables

**Table 1:** Energy and nutrient composition of fish meal (FM), infertile eggs (IFE), and a mixture of hatchery by-products (MIX)

Items	FM	IFE	MIX
AME_n_^1)^ (kcal/kg)	3,120	4,299	3,321
Nutrients^2)^			
DM (%)	90.4	98.3	98.0
CP (%)	68.5	34.2	31.4
Crude ash (%)	13.8	27.6	40.6
Calcium (%)	3.1	8.9	17.2
Phosphorus (%)	2.3	0.5	0.3
Total amino acid^3)^ (%)			
Lysine	4.6	2.3	1.9
Methionine+cysteine	2.2	1.8	1.8
Threonine	2.6	1.8	1.5
Tryptophan	0.6	0.4	0.4

AME_n_, nitrogen-corrected apparent metabolizable energy; DM, dry matter; CP, crude protein; AA, amino acid.

1) The value for AME_n_ of FM was adopted from INRA [35], whereas the values for AME_n_ of IFE and MIX were measured in our previous experiment [38].

2) Nutrient concentrations were analyzed using AOAC methods [39].

3) AA concentrations were analyzed for IFE and MIX, whereas AA concentrations for FM were adopted from INRA [35].

**Table 2:** Composition and nutrient concentration of the experimental diets from 3 d to 21 d of age

Items	Control	Inclusion levels of IFE (%)				Inclusion levels of MIX (%)			
	
		1.25	2.50	3.75	5.00	1.25	2.50	3.75	5.00
Ingredients (%)									
Corn	54.96	55.55	54.95	54.29	53.70	55.67	55.18	54.76	54.60
Soybean meal (46% CP)	30.89	29.49	30.36	31.22	32.08	29.53	30.47	31.35	32.21
Corn gluten meal	0.50	2.00	2.00	2.00	2.00	2.00	2.00	2.00	2.01
Tallow	3.98	3.14	2.95	2.79	2.60	3.24	3.14	3.03	2.80
FM	5.00	3.75	2.50	1.25	-	3.75	2.50	1.25	-
IFE	-	1.25	2.50	3.75	5.00	-	-	-	-
MIX	-	-	-	-	-	1.25	2.50	3.75	5.00
Limestone	1.27	1.11	0.94	0.78	0.60	0.83	0.38	0.00	0.00
Mono-dicalcium phosphate	0.72	0.86	0.98	1.10	1.22	0.87	1.00	0.95	0.00
Potassium phosphate	0.30	0.30	0.30	0.30	0.30	0.30	0.30	0.47	1.49
Potassium carbonate	0.59	0.65	0.63	0.62	0.61	0.64	0.63	0.53	0.00
L-Lysine sulfate (54%)	0.27	0.37	0.38	0.40	0.41	0.38	0.39	0.41	0.42
DL-methionine (88%)	0.41	0.39	0.38	0.38	0.37	0.39	0.38	0.38	0.37
L-threonine (98%)	0.10	0.10	0.10	0.10	0.09	0.11	0.10	0.10	0.10
L-tryptophan (19.6%)	0.01	0.04	0.03	0.02	0.02	0.04	0.03	0.02	0.00
Salt	0.20	0.20	0.20	0.20	0.20	0.20	0.20	0.20	0.20
Choline chloride	0.10	0.10	0.10	0.10	0.10	0.10	0.10	0.10	0.10
Sodium bicarbonate	0.10	0.10	0.10	0.10	0.10	0.10	0.10	0.10	0.10
Cocciodiostat	0.10	0.10	0.10	0.10	0.10	0.10	0.10	0.10	0.10
Vitamin premix^1)^	0.25	0.25	0.25	0.25	0.25	0.25	0.25	0.25	0.25
Mineral premix^2)^	0.25	0.25	0.25	0.25	0.25	0.25	0.25	0.25	0.25
Total	100	100	100	100	100	100	100	100	100
Calculated energy and nutrient concentration									
AME_n_ (kcal/kg)	3,050	3,050	3,050	3,050	3,050	3,050	3,050	3,050	3,050
CP (%)	22.25	22.25	22.25	22.25	22.25	22.25	22.25	22.25	22.25
Total lysine (%)	1.37	1.37	1.37	1.37	1.37	1.37	1.37	1.37	1.37
Total methionine+cysteine (%)	1.04	1.04	1.04	1.04	1.04	1.04	1.04	1.04	1.04
Total threonine (%)	0.93	0.93	0.93	0.93	0.93	0.93	0.93	0.93	0.93
Total tryptophan (%)	0.26	0.26	0.26	0.26	0.26	0.26	0.26	0.26	0.26
Ca (%)	1.00	1.00	1.00	1.00	1.00	1.00	1.00	1.00	1.00
Available phosphorus (%)	0.45	0.45	0.45	0.45	0.45	0.45	0.45	0.45	0.45
Potassium (%)	1.18	1.18	1.18	1.18	1.18	1.18	1.18	1.18	1.18

CP, crude protein; FM, fish meal; IFE, infertile eggs; MIX, a mixture of hatchery by-products; AME_n_, nitrogen-corrected apparent metabolizable energy.

1) Provided per kg of the complete diet: vitamin A, 10,000 IU (retinyl acetate); vitamin D_3_, 4,500 IU; vitamin K_3_, 3.0 mg (menadione dimethpyrimidinol); vitamin B_1_, 2.50 mg; vitamin B_2_, 6.50 mg; vitamin B_6_, 3.20 mg; vitamin B_12_, 18.0 μg; folic acid, 1.9 mg; biotin, 180 μg; niacin, 60 mg.

2) Provided per kg of the complete diet: iron, 72 mg (FeSO_4_); zinc, 120 mg (ZnSO_4_); manganese, 144.0 mg (MnO); copper, 19.0 mg (CuSO_4_); cobalt, 1,200 μg (CoSO_4_); selenium, 360 μg (Na_2_SeO_3_); iodine, 1.5 mg [Ca(IO_3_)_2_].

**Table 3:** Composition and nutrient concentration of the experimental diets from 22 d to 35 d of age

Items	Control	Inclusion levels of IFE (%)				Inclusion levels of MIX (%)			
	
		1.25	2.50	3.75	5.00	1.25	2.50	3.75	5.00
Ingredients (%)									
Corn	63.00	62.46	61.80	61.19	60.62	62.54	62.19	61.79	60.80
Soybean meal (46% CP)	21.84	22.68	23.59	24.43	25.27	22.76	23.63	24.54	25.54
Corn gluten meal	2.00	2.00	2.00	2.00	2.00	2.00	2.00	2.00	2.00
Tallow	3.71	3.49	3.32	3.14	2.94	3.60	3.45	3.32	3.43
FM	5.00	3.75	2.50	1.25	-	3.75	2.50	1.25	-
IFE	-	1.25	2.50	3.75	5.00	-	-	-	-
MIX	-	-	-	-	-	1.25	2.50	3.75	5.00
Limestone	1.16	0.97	0.81	0.63	0.47	0.70	0.24	0.00	0.00
Mono-dicalcium phosphate	0.58	0.70	0.81	0.95	1.05	0.70	0.81	0.45	0.00
Potassium phosphate	0.30	0.30	0.30	0.30	0.30	0.30	0.30	0.78	1.50
Potassium carbonate	0.66	0.65	0.64	0.63	0.62	0.65	0.63	0.38	0.00
L-lysine sulfate (54%)	0.29	0.30	0.31	0.33	0.34	0.30	0.32	0.34	0.35
DL-methionine (88%)	0.32	0.32	0.31	0.30	0.30	0.32	0.31	0.30	0.30
L-threonine (98%)	0.06	0.06	0.05	0.05	0.05	0.06	0.06	0.05	0.05
L-tryptophan (19.6%)	0.08	0.07	0.06	0.05	0.04	0.07	0.06	0.05	0.03
Salt	0.20	0.20	0.20	0.20	0.20	0.20	0.20	0.20	0.20
Choline chloride	0.10	0.10	0.10	0.10	0.10	0.10	0.10	0.10	0.10
Sodium bicarbonate	0.10	0.10	0.10	0.10	0.10	0.10	0.10	0.10	0.10
Cocciodiostat	0.10	0.10	0.10	0.10	0.10	0.10	0.10	0.10	0.10
Vitamin premix^1)^	0.25	0.25	0.25	0.25	0.25	0.25	0.25	0.25	0.25
Mineral premix^2)^	0.25	0.25	0.25	0.25	0.25	0.25	0.25	0.25	0.25
Total	100	100	100	100	100	100	100	100	100
Calculated energy and nutrient concentration									
AME_n_ (kcal/kg)	3,150	3,150	3,150	3,150	3,150	3,150	3,150	3,150	3,150
CP (%)	19.50	19.50	19.50	19.50	19.50	19.50	19.50	19.50	19.50
Total lysine (%)	1.16	1.16	1.16	1.16	1.16	1.16	1.16	1.16	1.16
Total methionine+cysteine (%)	0.91	0.91	0.91	0.91	0.91	0.91	0.91	0.91	0.91
Total threonine (%)	0.78	0.78	0.78	0.78	0.78	0.78	0.78	0.78	0.78
Total tryptophan (%)	0.23	0.23	0.23	0.23	0.23	0.23	0.23	0.23	0.23
Ca (%)	0.90	0.90	0.90	0.90	0.90	0.90	0.90	0.90	0.98
Available phosphorus (%)	0.40	0.40	0.40	0.40	0.40	0.40	0.40	0.40	0.44
Potassium (%)	1.08	1.08	1.08	1.08	1.08	1.08	1.08	1.08	1.08

CP, crude protein; FM, fish meal; IFE, infertile eggs; MIX, a mixture of hatchery by-products; AME_n_, nitrogen-corrected apparent metabolizable energy.

1) Provided per kg of the complete diet: vitamin A, 10,000 IU (retinyl acetate); vitamin D_3_, 4,500 IU; vitamin K_3_, 3.0 mg (menadione dimethpyrimidinol); vitamin B_1_, 2.50 mg; vitamin B_2_, 6.50 mg; vitamin B_6_, 3.20 mg; vitamin B_12_, 18.0 μg; folic acid, 1.9 mg; biotin, 180 μg; niacin, 60 mg.

2) Provided per kg of the complete diet: iron, 72 mg (FeSO_4_); zinc, 120 mg (ZnSO_4_); manganese, 144.0 mg (MnO); copper, 19.0 mg (CuSO_4_); cobalt, 1,200 μg (CoSO_4_); selenium, 360 μg (Na_2_SeO_3_); iodine, 1.5 mg [Ca(IO_3_)_2_].

**Table 4:** Effect of increasing inclusion levels of infertile eggs (IFE) as a replacement of fish meal (FM) in diets on growth performance of broiler chickens^1)^,^2)^

Items	Inclusion levels of IFE^3)^ (%)					SEM	p-value^4)^		
	
	0.00	1.25	2.50	3.75	5.00		T	L	Q
BWG (kg)	2.01	2.05	2.02	1.99	2.03	0.045	0.92	0.88	0.94
FI (kg)	3.05	3.08	3.07	3.05	3.08	0.049	0.98	0.72	0.95
FCR (kg/kg)	1.51	1.50	1.53	1.53	1.52	0.024	0.91	0.56	0.81

SEM, standard error of the mean; BWG, body weight gain; FI, feed intake; FCR, feed conversion ratio.

1) Data are least squares means of 8 observations per treatment.

2) Diets were fed to birds for 32 d from 3 to 35 d of age.

3) IFE was included at replacing levels of 0%, 25%, 50%, 75%, or 100% with 5.0% FM in the basal diet.

4) T, overall effects of treatments; L, linear effects of increasing replacement levels of IFE in diets; Q, quadratic effects of increasing replacement levels of IFE in diets.

**Table 5:** Effect of increasing inclusion levels of a mixture of hatchery by-products (MIX) as a replacement of fish meal (FM) in diets on growth performance of broiler chickens^1)^,^2)^

Items	Inclusion levels of MIX^3)^ (%)					SEM	p-value^4)^		
	
	0.00	1.25	2.50	3.75	5.00		T	L	Q
BWG (kg)	2.01	1.93	2.06	2.03	1.97	0.042	0.23	0.90	0.46
FI (kg)	3.05	2.98	3.21	3.11	3.08	0.053	0.05	0.24	0.22
FCR (kg/kg)	1.51	1.54	1.56	1.53	1.56	0.019	0.43	0.17	0.65

SEM, standard error of the mean; BWG, body weight gain; FI, feed intake; FCR, feed conversion ratio.

1) Data are least squares means of 8 observations per treatment.

2) Diets were fed to birds for 32 d from 3 to 35 d of age.

3) MIX was included at replacing levels of 0%, 25%, 50%, 75%, or 100% with 5.0% FM in the basal diet.

4) T, overall effects of treatments; L, linear effects of increasing replacement levels of MIX in diets; Q, quadratic effects of increasing replacement levels of MIX in diets.

**Table 6:** Effect of increasing inclusion levels of infertile eggs (IFE) as a replacement of fish meal (FM) in diets on the plasma measurements of broiler chickens^1)^

Items	Inclusion levels of IFE^2)^ (%)					SEM	p-value^3)^		
	
	0.00	1.25	2.50	3.75	5.00		T	L	Q
Albumin (g/dL)	1.03	1.06	1.11	1.05	1.09	0.040	0.67	0.40	0.51
ALT (U/L)	1.79	1.71	2.31	2.00	1.69	0.531	0.91	0.96	0.49
AST (U/L)	338	362	335	323	310	32.9	0.85	0.37	0.64
Creatinine (mg/dL)	0.30	0.31	0.31	0.23	0.27	0.038	0.57	0.24	0.86
Glucose (mg/dL)	293	277	281	251	299	20.7	0.57	0.81	0.24
Total protein (g/dL)	3.14	3.06	3.12	3.22	2.89	0.150	0.62	0.48	0.41
Total glyceride (mg/dL)	22.03	23.04	29.88	34.74	21.21	4.340	0.17	0.47	0.07

SEM, standard error of the mean; ALT, alanine aminotransferase; AST, aspartate aminotransferase.

1) Data are least squares means of 8 observations per treatment.

2) IFE was included at replacing levels of 0%, 25%, 50%, 75%, or 100% with 5.0% FM in the basal diet.

3) T, overall effects of treatments; L, linear effects of increasing replacement levels of IFE in diets; Q, quadratic effects of increasing replacement levels of IFE in diets.

**Table 7:** Effect of increasing inclusion levels of a mixture of hatchery by-products (MIX) as a replacement of fish meal (FM) in diets on the plasma measurements of broiler chickens^1)^

Items	Inclusion levels of MIX^2)^ (%)					SEM	p-value^3)^		
	
	0.00	1.25	2.50	3.75	5.00		T	L	Q
Albumin (g/dL)	1.03	0.98	1.12	1.05	1.08	0.038	0.15	0.15	0.81
ALT (U/L)	1.79	1.33	1.78	1.56	1.86	0.459	0.92	0.79	0.62
AST (U/L)	338	322	371	385	306	37.2	0.54	0.99	0.26
Creatinine (mg/dL)	0.30	0.31	0.28	0.32	0.30	0.042	0.99	1.00	1.00
Glucose (mg/dL)	293	258	296	276	280	18.3	0.59	0.88	0.77
Total protein (g/dL)	3.14	2.96	3.09	2.77	3.05	0.147	0.42	0.43	0.40
Total glyceride (mg/dL)	22.03	23.69	22.35	36.83	28.69	5.313	0.26	0.12	0.85

SEM, standard error of the mean; ALT, alanine aminotransferase; AST, aspartate aminotransferase.

1) Data are least squares means of 8 observations per treatment.

2) MIX was included at replacing levels of 0%, 25%, 50%, 75%, or 100% with 5.0% FM in the basal diet.

3) T, overall effects of treatments; L, linear effects of increasing replacement levels of MIX in diets; Q, quadratic effects of increasing replacement levels of MIX in diets.

**Table 8:** Effect of increasing inclusion levels of infertile eggs (IFE) as a replacement of fish meal (FM) in diets on the relative organ weight of broiler chickens^1)^

Items^2)^ (%)	Inclusion levels of IFE^3)^ (%)					SEM	p-value^4)^		
	
	0.00	1.25	2.50	3.75	5.00		T	L	Q
Breast	21.0	19.3	22.1	20.4	19.9	0.65	0.05	0.59	0.44
Liver	3.0	2.7	2.8	2.6	2.5	0.23	0.63	0.15	0.84
Kidney	0.6	0.5	0.6	0.5	0.6	0.04	0.58	0.91	0.27
Small intestine	3.1	3.2	3.0	3.0	3.0	0.15	0.75	0.45	0.93
Gizzard	1.3	1.3	1.2	1.3	1.4	0.05	0.18	0.16	<0.05

SEM, standard error of the mean.

1) Data are least squares means of 8 observations per treatment.

2) Relative organ weight = [organ weight (g)/body weight (g)]×100.

3) IFE was included at replacing levels of 0%, 25%, 50%, 75%, or 100% with 5.0% FM in the basal diet.

4) T, overall effects of treatments; L, linear effects of increasing replacement levels of IFE in diets; Q, quadratic effects of increasing replacement levels of IFE in diets.

**Table 9:** Effect of increasing inclusion levels of a mixture of hatchery by-products (MIX) as a replacement of fish meal (FM) in diets on the relative organ weight of broiler chickens^1)^

Items^2)^ (%)	Inclusion levels of MIX^3)^ (%)					SEM	p-value^4)^		
	
	0.00	1.25	2.50	3.75	5.00		T	L	Q
Breast	21.0	20.3	21.7	20.4	20.0	0.54	0.19	0.26	0.34
Liver	3.0	2.7	2.4	2.5	2.7	0.22	0.44	0.25	0.13
Kidney	0.6	0.7	0.5	0.6	0.6	0.04	0.20	0.55	0.56
Small intestine	3.1	3.2	3.0	3.1	3.1	0.12	0.74	0.83	0.75
Gizzard	1.3	1.4	1.2	1.4	1.3	0.07	0.48	0.82	0.84

SEM, standard error of the mean.

1) Data are least squares means of 8 observations per treatment.

2) Relative organ weight = [organ weight (g)/body weight (g)]×100.

3) MIX was included at replacing levels of 0%, 25%, 50%, 75%, or 100% with 5.0% FM in the basal diet.

4) T, overall effects of treatments; L, linear effects of increasing replacement levels of MIX in diets; Q, quadratic effects of increasing replacement levels of MIX in diets.

**Table 10:** Effect of increasing inclusion levels of infertile eggs (IFE) as a replacement of fish meal (FM) in diets on the immune organ index of broiler chickens^1)^

Items^2)^	Inclusion levels of IFE^3)^ (%)					SEM	p-value^4)^		
	
	0.00	1.25	2.50	3.75	5.00		T	L	Q
Thymus index	2.62	2.36	2.96	2.38	2.30	0.379	0.72	0.60	0.56
Spleen index	1.54	1.43	1.31	1.48	1.44	0.228	0.97	0.83	0.62
Bursal index	1.25	1.24	1.17	1.33	1.29	0.152	0.96	0.72	0.77

SEM, standard error of the mean.

1) Data are least squares means of 8 observations per treatment.

2) Index = [organ weight (g)/body weight (g)]×1,000.

3) IFE was included at replacing levels of 0%, 25%, 50%, 75%, or 100% with 5.0% FM in the basal diet.

4) T, overall effects of treatments; L, linear effects of increasing replacement levels of IFE in diets; Q, quadratic effects of increasing replacement levels of IFE in diets.

**Table 11:** Effect of increasing inclusion levels of a mixture of hatchery by-products (MIX) as a replacement of fish meal (FM) in diets on the immune organ index of broiler chickens^1)^

Items^2)^	Inclusion levels of MIX^3)^ (%)					SEM	p-value^4)^		
	
	0	25	50	75	100		T	L	Q
Thymus index	2.62	3.01	3.60	3.48	3.50	0.349	0.26	0.05	0.28
Spleen index	1.54	1.22	1.43	1.53	1.85	0.270	0.60	0.29	0.26
Bursal index	1.25	1.41	1.28	1.68	1.41	0.158	0.34	0.25	0.58

SEM, standard error of the mean.

1) Data are least squares means of 8 observations per treatment.

2) Index = [organ weight (g)/body weight (g)]×1,000.

3) MIX was included at replacing levels of 0%, 25%, 50%, 75%, or 100% with 5.0% FM in the basal diet.

4) T, overall effects of treatments; L, linear effects of increasing replacement levels of MIX in diets; Q, quadratic effects of increasing replacement levels of MIX in diets.

**Table 12:** Effect of increasing inclusion levels of infertile eggs (IFE) as a replacement of fish meal (FM) in diets on meat quality of breast meat of broiler chickens^1)^

Items	Inclusion levels of IFE^2)^ (%)					SEM	p-value^3)^		
	
	0.00	1.25	2.50	3.75	5.00		T	L	Q
Meat color									
L*	45.0	46.2	46.4	47.3	48.9	0.98	0.09	<0.05	0.69
a*	4.5	3.4	3.3	3.8	2.3	0.54	0.11	<0.05	0.92
b*	11.5	11.9	11.3	12.2	11.7	0.78	0.94	0.76	0.90
pH									
1 h	6.23^b^	6.36^ab^	6.39^ab^	6.54^a^	6.40^ab^	0.067	<0.05	<0.05	0.09
24 h	6.03	5.94	5.96	6.05	5.96	0.037	0.18	0.85	0.59
WHC	72.3	78.2	75.5	75.9	72.4	3.72	0.77	0.86	0.27
TBARS (mg MDA/kg)	0.33	0.33	0.31	0.31	0.31	0.012	0.49	0.19	0.74

SEM, standard error of the mean; L*, lightness; a*, redness; b*, yellowness; WHC, water holding capacity; TBARS, thiobarbituric acid-reactive substances; MDA, malondialdehyde.

1) Data are least squares means of 8 observations per treatment.

2) IFE was included at replacing levels of 0, 25, 50, 75, or 100% with 5.0% FM in the basal diet.

3) T, overall effects of treatments; L, linear effects of increasing replacement levels of IFE in diets; Q, quadratic effects of increasing replacement levels of IFE in diets.

a,b Means with different superscripts within a row are different (p<0.05).

**Table 13:** Effect of increasing inclusion levels of a mixture of hatchery by-products (MIX) as a replacement of fish meal (FM) in diets on meat quality of breast meat of broiler chickens^1)^

Items	Inclusion levels of MIX^2)^ (%)					SEM	p-value^3)^		
	
	0.00	1.25	2.50	3.75	5.00		T	L	Q
Meat color									
L*	45.0	46.7	46.1	45.8	45.6	0.92	0.77	0.90	0.31
a*	4.5	4.2	4.4	4.0	3.6	0.54	0.79	0.25	0.72
b*	11.5	12.6	12.3	12.0	11.8	0.59	0.68	0.97	0.22
pH									
1 h	6.23	6.36	6.39	6.34	6.40	0.051	0.14	<0.05	0.25
24 h	6.03	5.92	5.97	5.91	5.91	0.036	0.08	<0.05	0.48
WHC	72.3	75.2	74.5	73.3	74.1	3.50	0.98	0.87	0.72
TBARS (mg MDA/kg)	0.33	0.34	0.32	0.30	0.30	0.010	0.06	<0.05	0.41

SEM, standard error of the mean; L*, lightness; a*, redness; b*, yellowness; WHC, water holding capacity; TBARS, thiobarbituric acid-reactive substances; MDA, malondialdehyde.

1) Data are least squares means of 8 observations per treatment.

2) MIX was included at replacing levels of 0%, 25%, 50%, 75%, or 100% with 5.0% FM in the basal diet.

3) T, overall effects of treatments; L, linear effects of increasing replacement levels of MIX in diets; Q, quadratic effects of increasing replacement levels of MIX in diets.

**Table 14:** Effect of increasing inclusion levels of infertile eggs (IFE) as a replacement of fish meal (FM) in diets on the tibia characteristics of broiler chickens^1)^

Items	Inclusion levels of IFE^2)^ (%)					SEM	p-value^3)^		
	
	0.00	1.25	2.50	3.75	5.00		T	L	Q
Tibia ash (%)	52.7	52.0	52.1	52.3	52.7	0.50	0.76	0.87	0.20
Tibia calcium (%)	41.4	37.4	43.1	39.4	40.4	2.75	0.67	1.00	0.95
Tibia phosphorus (%)	16.1	16.1	17.2	15.5	15.9	1.11	0.84	0.76	0.62
Tibia breaking strength (kg)	35.3	38.6	34.3	41.5	36.0	2.87	0.40	0.64	0.59

SEM, standard error of the mean.

1) Data are least squares means of 8 observations per treatment.

2) IFE was included at replacing levels of 0%, 25%, 50%, 75%, or 100% with 5.0% FM in the basal diet.

3) T, overall effects of treatments; L, linear effects of increasing replacement levels of IFE in diets; Q, quadratic effects of increasing replacement levels of IFE in diets.

**Table 15:** Effect of increasing inclusion levels of a mixture of hatchery by-products (MIX) as a replacement of fish meal (FM) in diets on the tibia characteristics of broiler chickens^1)^

Items	Inclusion levels of MIX^2)^ (%)					SEM	p-value^3)^		
	
	0.00	1.25	2.50	3.75	5.00		T	L	Q
Tibia ash (%)	52.7	53.6	52.3	53.7	52.4	0.52	0.18	0.81	0.41
Tibia calcium (%)	41.4	39.7	42.8	40.6	42.0	2.90	0.96	0.83	0.94
Tibia phosphorus (%)	16.1	15.2	15.6	15.0	15.0	1.01	0.92	0.45	0.82
Tibia breaking strength (kg)	35.3	40.4	39.0	37.8	42.8	2.47	0.29	0.12	0.98

SEM, standard error of the mean.

1) Data are least squares means of 8 observations per treatment.

2) MIX was included at replacing levels of 0%, 25%, 50%, 75%, or 100% with 5.0% FM in the basal diet.

3) T, overall effects of treatments; L, linear effects of increasing replacement levels of MIX in diets; Q, quadratic effects of increasing replacement levels of MIX in diets.
